# Association of the gallbladder or biliary diseases with dipeptidyl peptidase 4 inhibitors in patients with type 2 diabetes: a meta-analysis of randomized controlled trials

**DOI:** 10.1186/s13098-022-00924-8

**Published:** 2022-10-21

**Authors:** Meng Yu, Zheng Yang, Chongxin Chen, Yuhuan Lv, Linyu Xiang, Subei Zhao, Rong Li

**Affiliations:** Department of Endocrinology, The First Affiliated Hospital of Chongqing Medical University, YouYi Road 1#, Yuzhong District, Chongqing, 400016 China

## Abstract

**Background:**

Previous studies have shown inconsistent conclusions regarding the association between incretin-based therapies and the risk of developing gallbladder or biliary diseases. We conducted a meta-analysis to evaluate the risk of gallbladder or biliary diseases associated with dipeptidyl peptidase 4 inhibitors (DPP4i) in patients with type 2 diabetes.

**Methods:**

The PubMed, Embase, Cochrane Library, and ClinicalTrials.gov databases were searched (from inception up to March 14, 2022) for published randomized controlled trials (RCTs) that compared DPP4i with placebo or other glucose-lowering drugs in patients with type 2 diabetes. The outcomes of interest were cholecystitis, cholangitis, cholelithiasis, bile duct stones, and biliary colic. Relative risks (RRs) and 95% confidence intervals (CI) were pooled using a random-effects model. Subgroup analyses were performed according to patient age, trial duration, and types of DPP4i.

**Results:**

In total, 97,150 participants from 75 eligible RCTs were included in the meta-analysis. DPP4i were associated with an increased risk of composite of gallbladder or biliary diseases (RR 1.20 [95% CI 1.01–1.42]) and cholecystitis (RR 1.38 [95% CI 1.08–1.75]). Among all included trials, DPP4i showed no association with the following manifestations of gallbladder or biliary diseases: cholelithiasis (RR 1.00 [95% CI 0.76–1.32]), cholangitis (RR 0.81 [95% CI 0.39–1.66]), bile duct stones (RR 1.08 [95% CI 0.57–2.05]), and biliary colic (RR 0.72 [95% CI 0.23–2.25]). Subgroup analyses showed that DPP4i were associated with a higher risk of cholecystitis in older patients (RR 1.37 [95% CI 1.03–1.83]) compared with younger patients (RR 1.08 [95% CI 0.89–2.18]) and in those with a longer duration of drug use (RR 1.43 [95% CI 1.08–1.89]) compared with shorter use (RR 1.23 [95% CI 0.74–2.03]).

**Conclusions:**

This systematic review and meta-analysis of RCTs found that the use of DPP4i was associated with an increased risk of cholecystitis, especially in patients of advanced age or in those who were exposed to the drugs for a long period of time.

**Supplementary Information:**

The online version contains supplementary material available at 10.1186/s13098-022-00924-8.

## Introduction

Dipeptidyl peptidase 4 inhibitors (DPP4i) are a class of oral hypoglycemic agents that have a favorable safety profile, do not cause hypoglycemia or weight gain, and do not require dose escalation. These agents have become firmly established in treatment algorithms and national guidelines for the management of type 2 diabetes. The basis for this approach lies with the finding that DPP4 has a key role in determining the clearance of incretins [1], which are gut-derived hormones that belong to the glucagon superfamily and are released after the intake of nutrients (mainly glucose and fats) [2]. As such, glucagon-like peptide-1 receptor agonists (GLP-1RAs) and DPP4i are included among incretin-based therapies [3].

However, regulatory concerns have been raised over whether incretin-based therapies are associated with a potentially elevated risk of gallbladder and biliary diseases. A population-based cohort study linked to the United Kingdom Clinical Practice Research Database found that the use of GLP-1RAs was associated with increased risks of cholelithiasis, cholecystitis, and cholangitis [4]. A post hoc analysis of the LEADER trial also found the use of GLP1-RAs as being associated with increased risks of gallbladder stone and cholecystitis development [5]. Additionally, a recent meta-analysis indicated that GLP-1RAs indeed increase the risk of gallbladder or biliary diseases [6]. Both DPP4i and GLP-1RAs have similar mechanisms of action, in that they induce the activation of GLP-1R [7]. However, whether patients taking DPP4i do indeed have a risk of developing gallbladder and biliary diseases remains controversial. EudraVigilance lists 200 serious adverse drug reactions associated with cholecystitis and the use of DPP4i [8]. However, it has been indicated in a population-based cohort study that the use of DPP4i is not associated with an increased risk of bile duct and gallbladder disease [4].

These inconsistencies in study conclusions prompted us to design and perform a meta-analysis to evaluate the potential risk of bile duct and gallbladder events associated with DPP4i in patients with type 2 diabetes. Data from randomized controlled trials (RCTs) were used for this review and meta-analysis.

## Methods

This meta-analysis was aligned with the Preferred Reporting Items for Systematic Reviews and Meta-Analyses (PRISMA) guidelines [9]. The protocol was registered in The International Prospective Register of Systematic Reviews (PROSPERO) (no.CRD42020155286).

### Data sources and searches

We searched PubMed, Embase, Cochrane Library, and ClinicalTrials.gov databases from inception up to March 14, 2022. RCTs that involved DPP4i were included without the restriction of language. Medical subject headings and free-text terms were used to identify the related articles. Details of search strategies were provided in Additional file 1. We also extracted data about adverse events related to the selected trials from ClinicalTrials.gov.

### Study selection

**Fig. 1:**
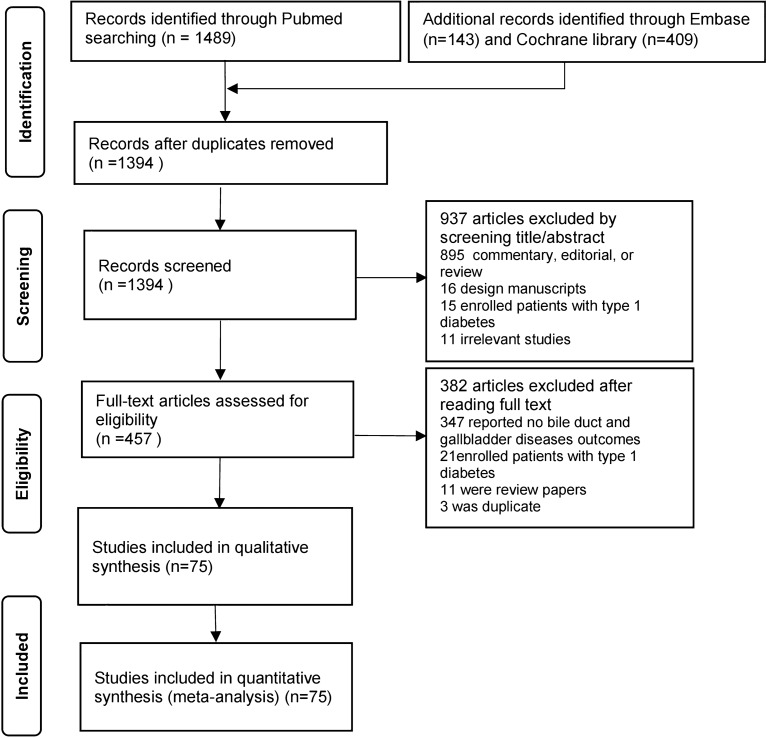
Study selection

We included all RCTs that reported gallbladder and biliary events during treatment with DPP4i and that had a control group, irrespective of the type of control substance used (placebo or active drug control). The gallbladder and biliary events of interest were cholecystitis, cholangitis, cholelithiasis, bile duct stones, and biliary colic. The schema of study selection is summarized in Fig. 1.

### Data extraction and quality evaluation

**Fig. 2:**
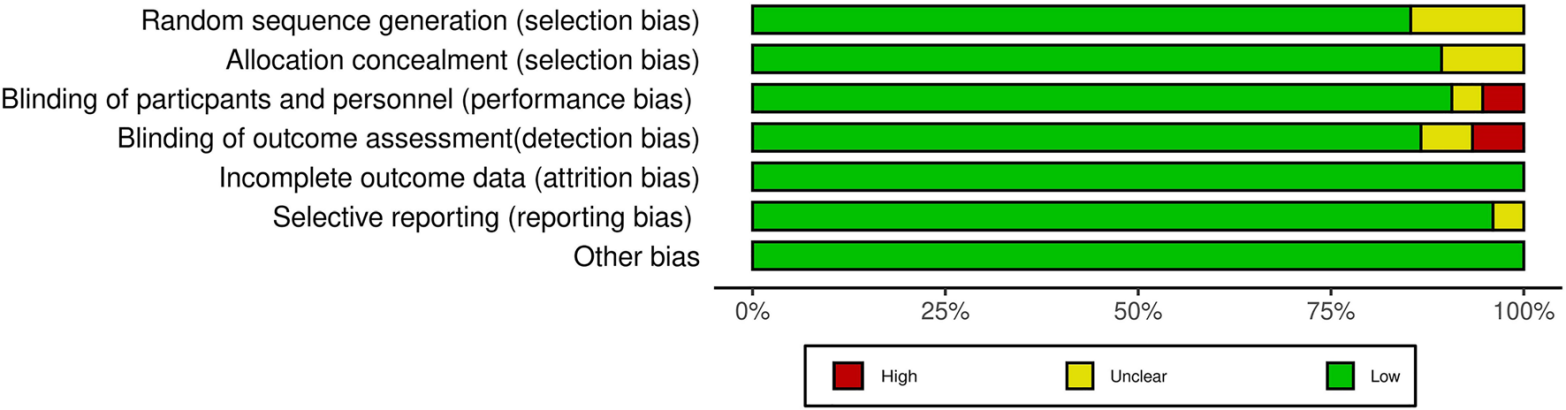
Risk of bias of included studies

For each study included in our analysis, data on the registration number, drug received by the control group (placebo, no additional treatment, or alternative oral hypoglycemic agent), background therapy, study duration, and treatment information (regimen and dose) were retrieved. Moreover, baseline characteristics (total number of patients and mean age) were collected for the DPP4i group and control group, respectively (Additional file 2). According to the Cochrane risk of bias tool (outlined in Chapter 8 of the *Cochrane Handbook for Systematic Reviews of Interventions* version 6.3) [10], we classified each RCT as having low, high, or unclear risk based on the following criteria: random sequence generation (selection bias), allocation concealment (selection bias), blinding (performance bias and detection bias), incomplete outcome data (attrition bias), and selective reporting (reporting bias) (Additional file 2), see Fig. 2 for an overview of the risks.

Study selection, data extraction, and quality assessment were conducted by two independent investigators (M.Y. and Z.Y.) with any disagreement resolved by consensus.

### Statistical analyses

Estimates from each study were combined for random-effects analysis using the Mantel–Haenszel method, with the relative risk (RR) and 95% confidence intervals (CIs) determined. The outcomes of interest were strictly identified using preferred terms from the *Medical Dictionary for Regulatory Activities* (MedDRA version 21.0). To assess whether the results were impacted by study characteristics (effect modifiers), the median age were used as cut-off points for age subgroups, where the treatment effect on the outcome was compared in the following subgroups: subclass of medicines, duration of follow-up (< 26 vs. ≥ 26 vs. ≥ 52 weeks), patient age (< 60 vs. ≥ 60 years old). Publication bias was assessed by constructing a funnel plot, the symmetry of which was assessed with Egger’s test. Inter-study heterogeneity was assessed using the I^2^ index and Cochran’s Q test. I^2^ values of lower than 25% indicated low heterogeneity, values of 26–50% indicated moderate heterogeneity, and values greater than 50% indicated high heterogeneity, and Cochran’s Q statistic p values of below 0.05 were considered indicators for significant heterogeneity.

Statistical analyses were primarily performed by using the STATA statistical software package (version 12.0).

## Results

In this meta-analysis, data were collected from 75 eligible trials involving 97,150 patients in total (median sample size: 700; range: 71–16,492 individuals). With regard to the type of controls used, 42 trials used placebos and 33 trials used active hypoglycemic agents. The median follow-up time was 26 weeks (range: 14–433 weeks). Reported events included cholecystitis (n = 264), cholangitis (n = 27), cholelithiasis (n = 194), bile duct stones (n = 36), and biliary colic (n = 13). The study information (trial name, NCT number, sample size, patient characteristics, and treatment information) are provided in Additional file 3.

**Fig. 3:**
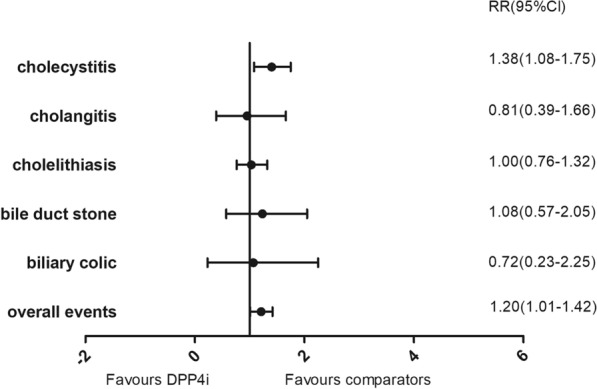
Risk of gallbladder or biliary diseases in DPP4i group compared with placebo and active comparators overall. Gallbladder or biliary diseases include cholecystitis, cholangitis, cholelithiasis, bile duct stone and biliary colic

In the analysis of all 75 trials, where the control group was placebo and active hypoglycemic agents overall, DPP4i were associated with a slightly increased risk of gallbladder or biliary diseases (RR 1.20 [95% CI 1.01–1.42]). In specific manifestations of gallbladder or biliary diseases: DPP4i were associated with an increased risk of cholecystitis (RR 1.38 [95% CI 1.08–1.75]), whereas they were not associated with increased risks of cholelithiasis (RR 1.00 [95% CI 0.76–1.32]), cholangitis (RR 0.81 [95% CI 0.39–1.66]), bile duct stones (RR 1.08 [95% CI 0.57–2.05]), and biliary colic (RR 0.72 [95% CI 0.23–2.25]) (Fig. 3).

**Fig. 4:**
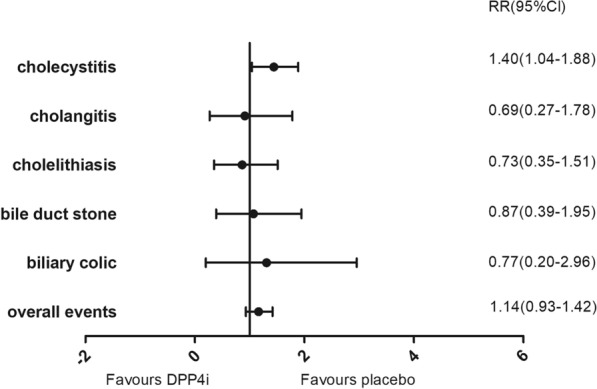
Risk of gallbladder or biliary diseases in DPP4i compared with placebo. Gallbladder or biliary diseases include cholecystitis, cholangitis, cholelithiasis, bile duct stone and biliary colic

We performed a separate analysis of all 42 trials that used placebo controls, whereupon no differences between DPP4i and placebos were found in terms of overall gallbladder or biliary disease risk (RR 1.14 [95% CI 0.93–1.42]). DPP4i were still associated with an increased risk of cholecystitis (RR 1.40 [95% CI 1.04–1.88]). Additionally, any apparent increased risks of cholelithiasis (RR 0.73 [95% CI 0.35–1.50]), cholangitis (RR 0.69 [95% CI 0.27–1.78]), bile duct stones (RR 0.87 [95% CI 0.39–1.95]), and biliary colic (RR 0.77 [95% CI 0.20–2.96]) did not reach statistical significance (Fig. 4).

**Fig. 5:**
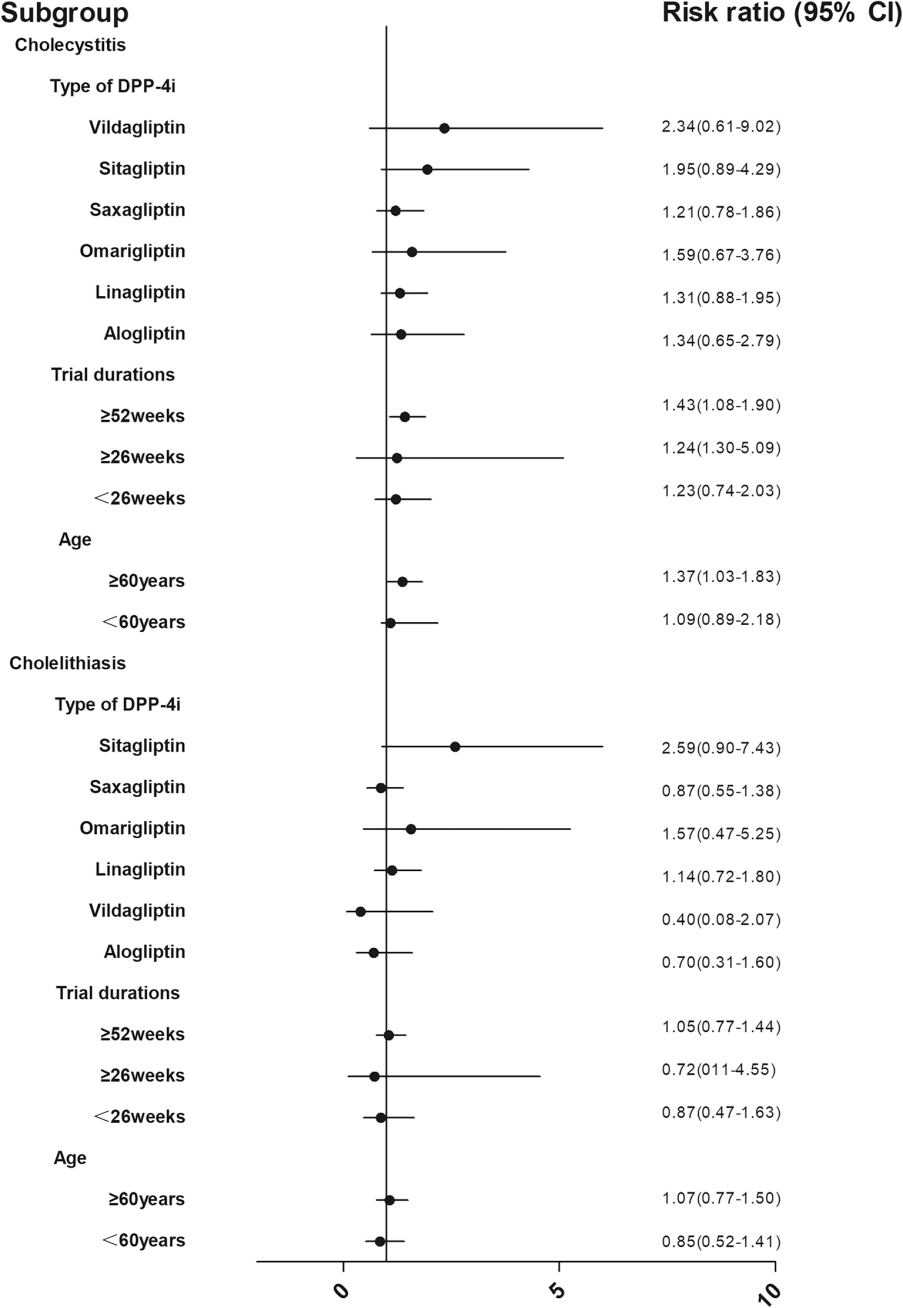
Subgroup analysis of DPP4i effects on cholecystitis and cholelithiasis events. Subgroup analysis were stratified by subclass of medicines, short versus long duration of follow-up (< 26 vs  ≥ 26 weeeks vs  ≥ 52 weeeks), age (< 60 vs  ≥ 60 years old)

For the subgroup analysis stratifying the studies according to patient age, trial duration, and drug subclass, the median age and follow-up time were selected as the cut-off points. Owing to an insufficient number of event reports, we only performed subgroup analysis for the risks of cholecystitis and cholelithiasis. In this regard, DPP4i were associated with a higher risk of cholecystitis in older patients (RR 1.37 [95% CI 1.03–1.83]) compared with younger patients (RR 1.09 [95% CI 0.89–2.18]) and in those with a longer duration (≥ 52 weeks) of drug use (RR 1.43 [95% CI 1.08–1.89]) compared with shorter exposure: ≥ 26 weeks (RR 1.24 [95% CI 0.30–5.09]), < 26 weeks(RR 1.22 [95% CI 0.74–2.03]). When the subgroup was stratified according to drug subclass, we found no significant heterogeneity of the DPP4i agents. With regard to risks of cholelithiasis, no significant heterogeneity was found in each subgroup analysis according to patient age, trial duration, or DPP4 inhibitor agents (Fig. 5).

Inter-study heterogeneity was assessed with the I^2^ index and Cochran’s Q test. The overall I^2^ ranges from 0.0% to 12.5%, P value ranges from 0.330 to 0.987, no significant inter-study statistical heterogeneity was identified (Additional file 4). Meanwhile, publication bias was assessed by constructing a funnel plot (Additional file 5) and Begg and Egger tests were carried out (Begg: p = 0.913, Egger: P = 0.698). The funnel plots indicated that the p value indicated that there was no obvious publication bias.

## Discussion

In the present meta-analysis of the association between DPP4i and risk of gallbladder or biliary diseases, we investigated 75 RCTs involving 97,150 participants with type 2 diabetes. Our findings revealed that the use of DPP4i was associated with the risk of cholecystitis. In the subgroup analysis, the pooled RR of cholecystitis was influenced by the patient age and follow-up duration. That is, the risk of cholecystitis associated with DPP4i was concentrated in the group of patients of advanced age or in those who had been exposed to the drugs for a long period of time. However, the use of DPP4i did not increase the risks of cholelithiasis, cholangitis, bile duct stones, and biliary colic.

Current studies suggest that DPP4 inhibitor use increases the risk of cholecystitis, which is consistent with our present results. A previous European Medicines Agency Assessment Report also suggested a strong association between DPP4 inhibitor use and the risk of cholecystitis [8]. According to another meta-analysis, the use of DPP4i was associated with a 43% increased risk of cholecystitis [11]. Compared with this article, we have several differences and new ideas. First, there are several papers linking glucose control and insulin sensitivity to biliary diseases [12, 13]. It is theoretically possible that non-incretin therapies could reduce the risk of biliary diseases when compared with “placebo therapy”; therefore, the apparent “increased rate” associated with DPP4i therapies could be an artifact. To exclude this effect, a separate placebo-controlled group was tested. Second, we focused on the subgroup analyses of patient age and duration of drug use. We found that the cholecystitis risk associated with DPP4i was greater in patients of advanced age (≥ 60 years old) or in those who had experienced long-time drug exposure (≥ 52 weeks). Furthermore, we also performed a drug-based subgroup analysis and found no agent heterogeneity. These results provide a theoretical basis for the clinical identification of high-risk patient groups and extend upon the previous work.

In the aging process, the wall of the gallbladder gradually becomes atrophied, causing the contractile function of the organ to decrease. These degenerative changes can lead to cholestasis. At the same time, the end of the common bile duct and the sphincter of Oddi become relaxed, rendering them prone to retrograde infection. These pathophysiological changes lead to older people being more susceptible to cholecystitis. This may explain the higher risk of cholecystitis in the elderly who use DPP4i [14]. Additionally, GLP-1 has been shown to enhance the proliferative and functional activities of cholangiocytes and prevent their apoptosis. Preclinical evidence suggests that GLP-1 reduces the production of bile acids. Furthermore, animal studies have shown that incretin-based therapy induces the prolongation of gallbladder refilling [15]. Other drugs, such as naltrexone, bupropion, and octreotide, have been demonstrated to increase the risk of gallbladder disorders through this mechanism. [16, 17]

GLP-1RAs would cause a loss of appetite and a significant improvement in body weight [18]. Rapid weight loss mobilizes cholesterol in the liver and adipose tissue and may increase cholesterol saturation in bile, which has been recognized as one of the risk factors for gallbladder disease [19, 20]. By contrast, DPP4i have no direct effect on gastrointestinal motility and lack the weight-loss effect of GLP-1RAs [21]. However, the use of DPP4i also increased the risk of acute cholecystitis. Remarkably, the enzyme DPP4 is also expressed on immune-related cells, and it seems to influence T-cell growth, differentiation, and activation. DPP4i may influence potential anti-inflammatory effects in addition to increasing the risk of cholecystitis [22, 23]. Accordingly, some studies have shown that DPP4i are associated with an increased risk of infections, such as nasopharyngitis and urinary tract infection [24, 25]. Interestingly, the enzyme DPP4 is located throughout the body, and it influences many other peptides and hormones, such as glucagon-like peptide 2 (GLP-2), glucose-dependent insulinotropic peptide (GIP), and polypeptide-YY (PYY), which have all been proven to exert effects on gallbladder motility [26]. PYY is a strong inhibitor of gallbladder emptying [27], GLP-2 receptor activation greatly increased the gallbladder volume in mice [28], and GIP was associated with cholecystokinin secretion in mouse models [29]. These could be the potential mechanisms through which DPP4i induce cholecystitis.

Several strengths of this article are worth mentioning. We conducted a prespecified subgroup analysis and found that advanced age and long-term drug exposure significantly increased the incidence of cholecystitis associated with DPP4i, which suggests that clinicians should be more cautious when using these types of inhibitors in these populations. From the clinical point of view, several gallbladder diseases (e.g., cholecystitis, cholangitis) were selected to explore the relationship between DPP4i and gallbladder disease. The severity and clinical significance of these diseases are sufficient to arouse the attention of clinicians.

However, there are several limitations of our study that are worth considering: (1) Since the occurrence of gallbladder and biliary diseases was not a primary or secondary outcome of the RCTs included in this article, reporting bias is possible. (2) In these patients with type 2 diabetes included in RTCs, other risk factors such as weight change, dietary habits, drinking history, obesity and dyslipidemia might contribute to the occurrence of gallbladder and biliary diseases. However, the above information is not fully accessible, the bias caused by these factors cannot be corrected. (3) Information on serious gallbladder and biliary events, such as hospitalization, surgery, or even death, is not fully available. (4) We only included RCTs. However, recent recommendations on the methodological quality of systematic reviews (AMSTAR2) suggest that if observational studies based on large population databases are combined with RCTs, the meta-estimates will generate precise estimates of intervention effects. It may be necessary to conduct special reviews of observational studies in the future.

## Conclusions

The findings of this systematic review and meta-analysis indicate that physicians should be concerned about the increased risk of cholecystitis associated with DPP4i use, especially in patients of advanced age and in those who have been exposed to the drugs for a long time. Additionally, future trials should prespecify gallbladder and biliary diseases as being potential adverse events and fully test for and report on these outcomes.

## Supplementary Information

**Additional file 1. **Search strategies.

**Additional file 2. **Risk of bias for each included study.

**Additional file 3. **Characteristics of the 75 studies included in the meta-analysis.

**Additional file 4. **Overall heterogeneity levels for each outcomes.

**Additional file 5. **Funnel plot for the meta-analysis of the association between DPP4i and risk of gallbladder or biliary diseases.

## Data Availability

All data generated or analysed during this study are included in this published article (and its supplementary information files).
