# Supplementary material for: Association of the gallbladder or biliary diseases with dipeptidyl peptidase 4 inhibitors in patients with type 2 diabetes: a meta-analysis of randomized controlled trials
Source: Diabetol Metab Syndr. 2022 Oct 21;14:153. doi: 10.1186/s13098-022-00924-8 (PMC9585736; doi:10.1186/s13098-022-00924-8)
Supplement: Supplementary file 1 — Additional file 1. Search strategies. [file 13098_2022_924_MOESM1_ESM.doc]

**Supplementary appendix 1 Search Strategies.**

| PUBMED | (DPP-4[TIAB] OR dipeptidyl peptidase 4[TIAB] OR sitagliptin[TIAB] OR vildagliptin[TIAB] OR saxagliptin[TIAB] OR alogliptin[TIAB] OR linagliptin[TIAB] OR teneligliptin[TIAB] OR anagliptin[TIAB] OR gemigliptin[TIAB] OR trelagliptin[TIAB]) AND (diabetes mellitus, type 2[MH] ) AND (Randomized Controlled Trial[pt] OR Randomized Controlled Trial[pt]) |
| --- | --- |
| EMBASE | 1.dipeptidyl peptidase iv inhibit.mp. 2.exp dipeptidyl peptidase IV inhibitor/ 3.exp dipeptidyl peptidase IV inhibitor/  4.dpp 4 inhibitor.mp. 5.dpp4i.mp. 6.dpp iv inhibitor.mp. 7.dppiv inhibitor.mp. 8.exp alogliptin/ 9.exp gemigliptin/ 10. exp linagliptin/  11.exp saxagliptin/ 12.exp sitagliptin/ 13.exp vildagliptin/  14.exp dutogliptin/ 15.exp teneligliptin/ 16.exp anagliptin/  17.exp evogliptin/ 18.retagliptin.mp 19.omarigliptin.mp  20.gliptin.mp. 21.exp clinical trial/ or exp controlled clinical trial/ or exp randomized controlled trial/ 22.random*.mp. 23.database*.mp.  24.1 or 2 or 3 or 4 or 5 or 6 or 7 or 8 or 9 or 10 or 11 or 12 or 13 or 14 or 15 or 16 or 17 or 18 or 19 or 20  25.21 or 22 or 23  24 and 25 |
| ClinicalTrials.gov | (DPP-4 inhibitors OR dipeptidyl peptidase 4 inhibitor OR DPP-4 or dipeptidyl peptidase 4 OR sitagliptin OR vildagliptin OR saxagliptin OR alogliptin OR linagliptin OR teneligliptin OR anagliptin OR gemigliptin OR trelagliptin)  (Diabetes Mellitus, Type 2) |
