# Supplementary figures and images for: Association of the gallbladder or biliary diseases with dipeptidyl peptidase 4 inhibitors in patients with type 2 diabetes: a meta-analysis of randomized controlled trials
Source: Diabetol Metab Syndr. 2022 Oct 21;14:153. doi: 10.1186/s13098-022-00924-8 (PMC9585736; doi:10.1186/s13098-022-00924-8)

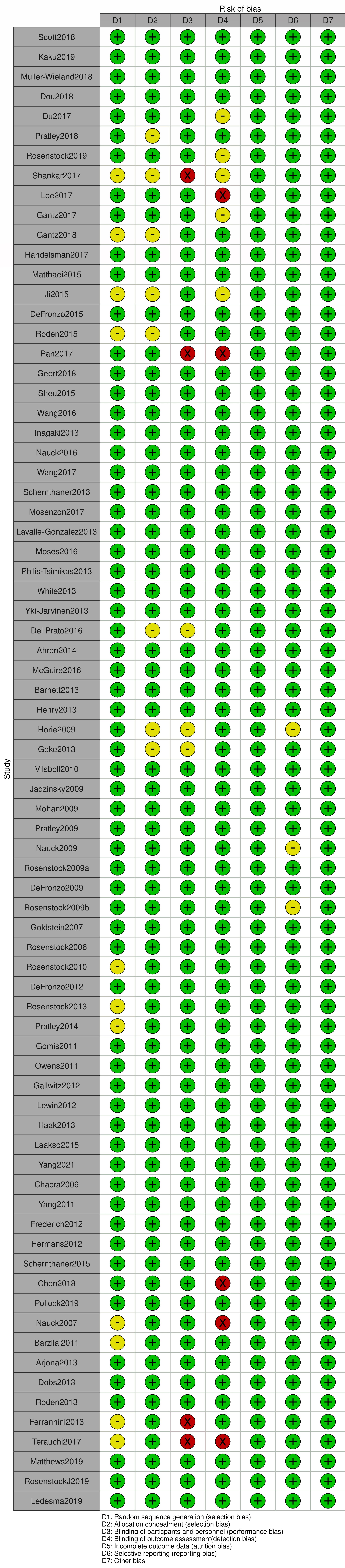

Supplement: Supplementary file 2 — Additional file 2. Risk of bias for each included study. [file 13098_2022_924_MOESM2_ESM.jpg]
