# Supplementary material for: Association of the gallbladder or biliary diseases with dipeptidyl peptidase 4 inhibitors in patients with type 2 diabetes: a meta-analysis of randomized controlled trials
Source: Diabetol Metab Syndr. 2022 Oct 21;14:153. doi: 10.1186/s13098-022-00924-8 (PMC9585736; doi:10.1186/s13098-022-00924-8)
Supplement: Supplementary file 3 — Additional file 3. Characteristics of the 75 studies included in the meta-analysis. [file 13098_2022_924_MOESM3_ESM.docx]

**Supplementary Appendix 3 Characteristics of the 75 studies included in the meta-analysis**

| **Study ID** | **No.NCT** | **Experiment treatments** | **Control treatments** | **Background medicine** | **Size of control/experi-ment group** | **Trial duration(w)** | **Age (yrs)** | **The num(%) of patients reporting each type of outcome events** | | | | |
| --- | --- | --- | --- | --- | --- | --- | --- | --- | --- | --- | --- | --- |
|  |  |  |  |  |  |  |  | **Cholecystitis** | **Cholangitis** | **cholelithiasis** | **bile duct stone** | **biliary colic** |
| **Scott2018^1^** | **NCT02532855** | **Sitagliptin** | **Dapagliflozin** | **Metformin ± sulfonylurea** | **307/306** | **26** | **67** | **0(0.00%)** | **1(0.16%)** | **0(0.00%)** | **0(0.00%)** | **0(0.00%)** |
| **Kaku2019^2^** | **NCT02489968** | **Linagliptin** | **Placebo** | **Empagliflozin+Metformin** | **223/224** | **24** | **57.2** | **0(0.00%)** | **1(0.22%)** | **0(0.00%)** | **0(0.00%)** | **0(0.00%)** |
| **Muller-Wieland2018^3^** | **NCT02471404** | **Saxagliptin** | **Dapagliflozin** | **Metformin+Dapagliflozin** | **312/313** | **52** | **58.4** | **0(0.00%)** | **0(0.00%)** | **2(0.32%)** | **0(0.00%)** | **0(0.00%)** |
| **Dou2018^4^** | **NCT02273050** | **Saxagliptin** | **Placebo** | **Metformin** | **429/210** | **24** | **50.1** | **2(0.31%)** | **0(0.00%)** | **1(0.16%)** | **0(0.00%)** | **0(0.00%)** |
| **Du2017^5^** | **NCT02243176** | **Saxagliptin** | **Acarbose** | **Metformin** | **241/244** | **24** | **55.6** | **1(0.21%)** | **0(0.00%)** | **1(0.21%)** | **0(0.00%)** | **0(0.00%)** |
| **Pratley2018^6^** | **NCT02099110** | **Sitagliptin** | **Ertugliflozin** | **Metformin ± Glimepiride** | **734/498** | **54** | **55** | **3(0.24%)** | **0(0.00%)** | **1(0.08%)** | **0(0.00%)** | **0(0.00%)** |
| **Rosenstock2019^7^** | **NCT01897532** | **Linagliptin** | **Placebo** | **OADs ± insulin** | **3494/3485** | **24** | **65.9** | **29(0.42%)** | **3(0.04%)** | **19(0.27%)** | **4(0.06%)** | **2(0.03%)** |
| **Shankar2017^8^** | **NCT01755156** | **Omarigliptin** | **Placebo/Glimepiride** | **Metformin** | **201/201** | **80** | **57.2** | **0(0.00%)** | **0(0.00%)** | **1(0.25%)** | **0(0.00%)** | **0(0.00%)** |
| **Lee2017^9^** | **NCT01704261** | **Omarigliptin** | **Placebo** | **Glimepiride + Metformin** | **153/153** | **24** | **57.8** | **1(0.33%)** | **0(0.00%)** | **0(0.00%)** | **0(0.00%)** | **0(0.00%)** |
| **Gantz2017^10^** | **NCT01703221** | **Omarigliptin** | **Placebo** | **\** | **166/82** | **24** | **60** | **0(0.00%)** | **0(0.00%)** | **0(0.00%)** | **1(0.40%)** | **0(0.00%)** |
| **Gantz2018^11^** | **NCT01703208** | **Omarigliptin** | **Placebo** | **OADs ± Insulin** | **2092/2100** | **234** | **63.6** | **19(0.45%)** | **2(0.05%)** | **8(0.19%)** | **1(0.02%)** | **0(0.00%)** |
| **Handelsman2017^12^** | **NCT01682759** | **Omarigliptin** | **Glimepiride** | **Metformin** | **375/375** | **54** | **57.7** | **1(0.13%)** | **0(0.00%)** | **0(0.00%)** | **0(0.00%)** | **0(0.00%)** |
| **Matthaei2015^13^** | **NCT01619059** | **Saxagliptin** | **Placebo** | **Dapagliflozin + Metformin** | **162/153** | **24** | **54.6** | **0(0.00%)** | **0(0.00%)** | **1(0.32%)** | **0(0.00%)** | **0(0.00%)** |
| **Ji2015^14^** | **NCT01438814** | **Linagliptin** | **Metformin** | **Metformin** | **344/345** | **14** | **53** | **1(0.15%)** | **0(0.00%)** | **0(0.00%)** | **0(0.00%)** | **0(0.00%)** |
| **DeFronzo2015^15^** | **NCT01422876** | **Linagliptin** | **Empagliflozin** | **±Metformin** | **812/551** | **52** | **55.4** | **2(0.15%)** | **0(0.00%)** | **2(0.15%)** | **0(0.00%)** | **0(0.00%)** |
| **Roden2015^16^** | **NCT01289990** | **Sitagliptin** | **Placebo** | **Metformin/Pioglitazone** | **223/676** | **76** | **55** | **3(0.33%)** | **0(0.00%)** | **0(0.00%)** | **0(0.00%)** | **0(0.00%)** |
| **Pan2017^17^** | **NCT01289119** | **Alogliptin** | **Placebo** | **Metformin/Pioglitazone** | **252/253** | **16** | **52.6** | **0(0.00%)** | **1(0.20%)** | **0(0.00%)** | **0(0.00%)** | **0(0.00%)** |
| **Geert2018^18^** | **NCT01243424** | **Linagliptin** | **Glimepiride** | **OADs** | **3023/3010** | **433** | **64** | **53(0.88%)** | **5(0.08%)** | **34(0.56%)** | **7(0.12%)** | **2(0.03%)** |
| **Sheu2015^19^** | **NCT01217073** | **Omarigliptin** | **Placebo/Metformin** | **±Metformin/Pioglitazone** | **405/80** | **66** | **55.1** | **0(0.00%)** | **0(0.00%)** | **1(0.21%)** | **0(0.00%)** | **0(0.00%)** |
| **Wang2016^20^** | **NCT01215097** | **Linagliptin** | **Placebo** | **Metformin ± Glimepiride** | **205/100** | **24** | **55.5** | **1(0.33%)** | **0(0.00%)** | **0(0.00%)** | **0(0.00%)** | **0(0.00%)** |
| **Inagaki2013^21^** | **NCT01204294** | **Linagliptin** | **SU/A-GI+Met** | **OADs** | **450/124** | **52** | **60.9** | **1(0.17%)** | **0(0.00%)** | **1(0.17%)** | **0(0.00%)** | **0(0.00%)** |
| **Nauck2016^22^** | **NCT01183013** | **Linagliptin** | **Pioglitazone** | **\** | **527/409** | **54** | **57.1** | **1(0.11%)** | **0(0.00%)** | **0(0.00%)** | **0(0.00%)** | **0(0.00%)** |
| **Wang2017^23^** | **NCT01177384** | **Sitagliptin** | **Placebo** | **Acarbose** | **191/189** | **24** | **57.15** | **2(0.53%)** | **0(0.00%)** | **0(0.00%)** | **1(0.26%)** | **0(0.00%)** |
| **Schernthaner2013^24^** | **NCT01137812** | **Sitagliptin** | **Canagliflozin** | **Metformin+Sulfonylurea** | **378/377** | **52** | **56.7** | **0(0.00%)** | **0(0.00%)** | **1(0.13%)** | **0(0.00%)** | **0(0.00%)** |
| **Mosenzon2017^25^** | **NCT01107886** | **Saxagliptin** | **Placebo** | **OADs ± Insulin** | **8280/8212** | **151** | **65** | **64(0.39%)** | **4(0.02%)** | **53(0.32%)** | **8(0.05%)** | **4(0.02%)** |
| **Lavalle-Gonzalez2013^26^** | **NCT01106677** | **Sitagliptin** | **Canagliflozin** | **Metformin** | **366/735** | **52** | **55.4** | **2(0.18%)** | **1(0.09%)** | **0(0.00%)** | **0(0.00%)** | **0(0.00%)** |
| **Moses2016^27^** | **NCT01076075** | **Sitagliptin** | **Placebo/Pioglitazone** | **Metformin+Sulfonylurea** | **210/212** | **54** | **54.9** | **0(0.00%)** | **0(0.00%)** | **1(0.24%)** | **0(0.00%)** | **0(0.00%)** |
| **Philis-Tsimikas2013^28^** | **NCT01046110** | **Sitagliptin** | **IDeg** | **OADs** | **228/226** | **26** | **55.7** | **0(0.00%)** | **0(0.00%)** | **1(0.22%)** | **0(0.00%)** | **0(0.00%)** |
| **White2013^29^** | **NCT00968708** | **Alogliptin** | **Placebo** | **OADs ± Insulin** | **2701/2679** | **77** | **60.9** | **16(0.30%)** | **2(0.04%)** | **16(0.30%)** | **2(0.04%)** | **1(0.02%)** |
| **Yki-Jarvinen2013^30^** | **NCT00954447** | **Linagliptin** | **Placebo** | **lnsulin ± OADs** | **631/630** | **52** | **60** | **2(0.16%)** | **0(0.00%)** | **1(0.08%)** | **0(0.00%)** | **0(0.00%)** |
| **Del Prato2016^31^** | **NCT00856284** | **Alogliptin** | **Sulfonylurea** | **Metformin** | **1751/869** | **104** | **55.4** | **6(0.23%)** | **0(0.00%)** | **2(0.08%)** | **1(0.04%)** | **0(0.00%)** |
| **Ahren2014^32^** | **NCT00838903** | **Sitagliptin** | **Placebo/Glimepiride** | **Metformin** | **302/408** | **156** | **54.5** | **1(0.14%)** | **0(0.00%)** | **2(0.28%)** | **1(0.14%)** | **0(0.00%)** |
| **McGuire2016^33^** | **NCT00790205** | **Sitagliptin** | **Placebo** | **OADs ± Insulin** | **7266/7274** | **261** | **65.4** | **1(0.01%)** | **0(0.00%)** | **1(0.01%)** | **1(0.01%)** | **2(0.01%)** |
| **Barnett2013^34^** | **NCT00757588** | **Saxagliptin** | **Placebo** | **Insulin ± Metformin** | **304/151** | **52** | **57.2** | **0(0.00%)** | **2(0.44%)** | **0(0.00%)** | **0(0.00%)** | **0(0.00%)** |
| **Henry2013^35^** | **NCT00722371** | **Sitagliptin** | **Pioglitazone** | **Metformin/Sulphonylurea** | **922/693** | **54** | **NA** | **0(0.00%)** | **0(0.00%)** | **1(0.06%)** | **0(0.00%)** | **0(0.00%)** |
| **Horie2009^36^** | **NCT00654381** | **Linagliptin** | **Voglibose** | **\** | **319/162** | **54** | **60** | **0(0.00%)** | **1(0.21%)** | **0(0.00%)** | **2(0.42%)** | **0(0.00%)** |
| **Goke2013^37^** | **NCT00575588** | **Saxagliptin** | **Glipizide** | **Metformin** | **428/430** | **52** | **57.55** | **2(0.23%)** | **0(0.00%)** | **2(0.23%)** | **0(0.00%)** | **1(0.12%)** |
| **Vilsboll2010^38^** | **NCT00395343** | **Sitagliptin** | **Placebo** | **OADs ± Insulin** | **322/319** | **24** | **57.8** | **1(0.16%)** | **0(0.00%)** | **1(0.16%)** | **0(0.00%)** | **0(0.00%)** |
| **Jadzinsky2009^39^** | **NCT00327015** | **Saxagliptin** | **Metformin** | **\** | **978/328** | **24** | **51.99** | **2(0.15%)** | **0(0.00%)** | **2(0.15%)** | **0(0.00%)** | **0(0.00%)** |
| **Mohan2009^40^** | **NCT00289848** | **Sitagliptin** | **Placebo** | **\** | **352/178** | **18** | **50.9** | **1(0.19%)** | **0(0.00%)** | **0(0.00%)** | **1(0.19%)** | **0(0.00%)** |
| **Pratley2009^41^** | **NCT00286468** | **Alogliptin** | **Placebo** | **Glibenclamide** | **401/99** | **26** | **57** | **1(0.20%)** | **0(0.00%)** | **0(0.00%)** | **0(0.00%)** | **0(0.00%)** |
| **Nauck2009^42^** | **NCT00286442** | **Alogliptin** | **Placebo** | **Metformin** | **420/104** | **26** | **55** | **0(0.00%)** | **0(0.00%)** | **1(0.19%)** | **0(0.00%)** | **0(0.00%)** |
| **Rosenstock2009^43^** | **NCT00286429** | **Alogliptin** | **Placebo** | **Insulin ± Metformin** | **260/129** | **26** | **55** | **2(0.51%)** | **0(0.00%)** | **0(0.00%)** | **0(0.00%)** | **0(0.00%)** |
| **DeFronzo2009^44^** | **NCT00121667** | **Saxagliptin** | **Placebo** | **Metformin** | **564/179** | **180** | **54.57** | **4(0.54%)** | **0(0.00%)** | **3(0.40%)** | **0(0.00%)** | **0(0.00%)** |
| **Rosenstock2009^45^** | **NCT00121641** | **Saxagliptin** | **Placebo** | **\** | **306/95** | **24** | **53.46** | **1(0.25%)** | **0(0.00%)** | **1(0.25%)** | **0(0.00%)** | **0(0.00%)** |
| **Goldstein2007^46^** | **NCT00103857** | **Sitagliptin** | **Metformin** | **\** | **668/540** | **104** | **53.4** | **1(0.08%)** | **1(0.08%)** | **0(0.00%)** | **1(0.08%)** | **0(0.00%)** |
| **Rosenstock2006^47^** | **NCT00086502** | **Sitagliptin** | **Placebo** | **Pioglitazone** | **175/178** | **24** | **56.25** | **1(0.28%)** | **0(0.00%)** | **0(0.00%)** | **0(0.00%)** | **0(0.00%)** |
| **Rosenstock2010^48^** | **NCT00395512** | **Alogliptin** | **Pioglitazone** | **\** | **164/163** | **26** | **52.6** | **1(0.30%)** | **0****(0.00%)** | **0(0.00%)** | **0(0.00%)** | **0(0.00%)** |
| **DeFronzo2012^49^** | **NCT00328627** | **Alogliptin** | **Placebo** | **Pioglitazone** | **257/129** | **26** | **54.4** | **1(0.26%)** | **0(0.00%)** | **1(0.26%)** | **0(0.00%)** | **1(0.26%)** |
| **Rosenstock2013^50^** | **NCT 00707993** | **Alogliptin** | **Glipizide** | **\** | **222/219** | **52** | **69.9** | **1(0.23%)** | **1(0.23%)** | **0(0.00%)** | **0(0.00%)** | **0(0.00%)** |
| **Pratley2014^51^** | **NCT 01023581** | **Alogliptin** | **Metformin** | **\** | **114/111** | **26** | **53.5** | **1(0.44%)** | **0(0.00%)** | **0(0.00%)** | **0(0.00%)** | **0(0.00%)** |
| **Gomis2011^52^** | **NCT00641043** | **Linagliptin** | **Placebo** | **Pioglitazone** | **259/130** | **24** | **57.5** | **0(0.00%)** | **1(0.26%)** | **0(0.00%)** | **0(0.00%)** | **1(0.26%)** |
| **Owens2011^53^** | **NCT00602472** | **Linagliptin** | **Placebo** | **Metformin/Sulphonylurea** | **792/263** | **24** | **58.1** | **1(0.09%)** | **0(0.00%)** | **0(0.00%)** | **0(0.00%)** | **1(0.09%)** |
| **Gallwitz B2012^54^** | **NCT00622284** | **Linagliptin** | **Glimepiride** | **Metformin** | **776/775** | **104** | **59.8** | **2(0.13%)** | **2(0.13%)** | **0(0.00%)** | **1(0.06%)** | **3(0.19%)** |
| **Lewin AJ2012^55^** | **NCT00819091** | **Linagliptin** | **Placebo** | **Sulphonylurea** | **161/87** | **12** | **56.9** | **0(0.00%)** | **0(0.00%)** | **0****(0.00%)** | **0(0.00%)** | **1(0.40%)** |
| **Haak T2013^56^** | **NCT00915772** | **Linagliptin** | **Metformin** | **\** | **171/170** | **78** | **55.8** | **1(0.29%)** | **0(0.00%)** | **0(0.00%)** | **0(0.00%)** | **1(0.29%)** |
| **Laakso M2015^57^** | **NCT01087502** | **Linagliptin** | **Glimepiride** | **\** | **113/112** | **52** | **66.6** | **0(0.00%)** | **0(0.00%)** | **0(0.00%)** | **0(0.00%)** | **0(0.00%)** |
| **Yang W2021^58^** | **NCT02897349** | **Linagliptin** | **Placebo** | **Insulin ± Metformin** | **104/102** | **24** | **58.7** | **1(0.49%)** | **0(0.00%)** | **0(0.00%)** | **0(0.00%)** | **1(0.49%)** |
| **Chacra AR2009^59^** | **NCT00313313** | **Saxagliptin** | **Placebo** | **Sulphonylurea** | **253/267** | **24** | **55.09** | **2(0.37%)** | **0(0.00%)** | **0(0.00%)** | **0(0.00%)** | **0(0.00%)** |
| **Yang W2011^60^** | **NCT00661362** | **Saxagliptin** | **Placebo** | **Metformin** | **283/287** | **24** | **54.05** | **1(0.18%)** | **0(0.00%)** | **0(0.00%)** | **0(0.00%)** | **0(0.00%)** |
| **Frederich R2012 ^61^** | **NCT00316082** | **Saxagliptin** | **Placebo** | **OADs ± Insulin** | **72/74** | **24** | **54.98** | **2(1.37%)** | **0(0.00%)** | **0(0.00%)** | **0(0.00%)** | **1(0.68%)** |
| **Hermans MP2012^62^** | **NCT01006590** | **Saxagliptin** | **Metformin** | **\** | **147/139** | **24** | **58.7** | **1(0.35%)** | **0(0.00%)** | **0(0.00%)** | **0(0.00%)** | **0(0.00%)** |
| **Schernthaner2015^63^** | **NCT01006603** | **Saxagliptin** | **Glimepiride** | **Metformin** | **359/359** | **52** | **72.6** | **1(0.14%)** | **0(0.00%)** | **0(0.00%)** | **0(0.00%)** | **0(0.00%)** |
| **Chen2018^64^** | **NCT02104804** | **Saxagliptin** | **Placebo** | **Insulin ± Metformin** | **234/231** | **24** | **59.1** | **0(0.00%)** | **1(0.22%)** | **0(0.00%)** | **0(0.00%)** | **0(0.00%)** |
| **Pollock2019^65^** | **NCT02547935** | **Saxagliptin** | **Dapagliflozin** | **\** | **152/145** | **28** | **64.4** | **0(0.00%)** | **0(0.00%)** | **0(0.00%)** | **0(0.00%)** | **0(0.00%)** |
| **Nauck2007^66^** | **NCT00094770** | **Sitagliptin** | **Glipizide** | **\** | **588/584** | **104** | **56.7** | **2(0.17%)** | **0(0.00%)** | **0(0.00%)** | **0(0.00%)** | **4(0.34%)** |
| **Barzilai2011^67^** | **NCT00305604** | **Sitagliptin** | **Placebo** | **\** | **102/104** | **24** | **71.9** | **0(0.00%)** | **1(0.49%)** | **0(0.00%)** | **0(0.00%)** | **0(0.00%)** |
| **Arjona2013^68^** | **NCT00509262** | **Sitagliptin** | **Glipizide** | **\** | **210/212** | **54** | **64.2** | **1(0.24%)** | **1(0.24%)** | **0(0.00%)** | **0(0.00%)** | **1(0.24%)** |
| **Dobs2013^69^** | **NCT00350779** | **Sitagliptin** | **Placebo** | **OADs ± Insulin** | **170/92** | **54** | **54.5** | **0(0.00%)** | **0(0.00%)** | **0(0.00%)** | **0(0.00%)** | **0(0.00%)** |
| **Roden2013^70^** | **NCT01177813** | **Sitagliptin** | **Placebo** | **\** | **223/229** | **24** | **55** | **2(0.44%)** | **1(0.22%)** | **0(0.00%)** | **0(0.00%)** | **0(0.00%)** |
| **Ferrannini2013^71^** | **NCT00881530** | **Sitagliptin** | **Metformin** | **\** | **56/56** | **78** | **58.6** | **2(1.79%)** | **0(0.00%)** | **0(0.00%)** | **0(0.00%)** | **1(0.89%)** |
| **Terauchi2017^72^** | **NCT01183104** | **Sitagliptin** | **Glimepiride** | **OADs** | **148/143** | **52** | **70.5** | **0(0.00%)** | **0(0.00%)** | **0(0.00%)** | **0(0.00%)** | **1(0.34%)** |
| **Matthews2019^73^** | **NCT01528254** | **vildagliptin** | **Placebo** | **Metformin** | **998/1001** | **260** | **54.3** | **7(0.35%)** | **3(0.15%)** | **0(0.00%)** | **1(0.05%)** | **2(0.10%)** |
| **RosenstockJ2019^74^** | **NCT02681094** | **Saxagliptin** | **Dapagliflozin** | **Metformin** | **295/293** | **24** | **56.7** | **0(0.00%)** | **0(0.00%)** | **0(0.00%)** | **0(0.00%)** | **1(0.17%)** |
| **Ledesma2019^75^** | **NCT02240680** | **Linagliptin** | **Placebo** | **\** | **52/50** | **52** | **72.4** | **0(0.00%)** | **1(0.98%)** | **0(0.00%)** | **0(0.00%)** | **0(0.00%)** |

1. Scott R, Morgan J, Zimmer Z, et al. A randomized clinical trial of the efficacy and safety of sitagliptin compared with dapagliflozin in patients with type 2 diabetes mellitus and mild renal insufficiency: The CompoSIT-R study. *Diabetes Obes Metab* 2018; **20**(12): 2876-84.

2. Kaku K, Haneda M, Tanaka Y, et al. Linagliptin as add-on to empagliflozin in a fixed-dose combination in Japanese patients with type 2 diabetes: Glycaemic efficacy and safety profile in a two-part, randomized, placebo-controlled trial. *Diabetes Obes Metab* 2019; **21**(1): 136-45.

3. Muller-Wieland D, Kellerer M, Cypryk K, et al. Efficacy and safety of dapagliflozin or dapagliflozin plus saxagliptin versus glimepiride as add-on to metformin in patients with type 2 diabetes. *Diabetes Obes Metab* 2018; **20**(11): 2598-607.

4. Dou J, Ma J, Liu J, et al. Efficacy and safety of saxagliptin in combination with metformin as initial therapy in Chinese patients with type 2 diabetes: Results from the START study, a multicentre, randomized, double-blind, active-controlled, phase 3 trial. *Diabetes Obes Metab* 2018; **20**(3): 590-8.

5. Du J, Liang L, Fang H, et al. Efficacy and safety of saxagliptin compared with acarbose in Chinese patients with type 2 diabetes mellitus uncontrolled on metformin monotherapy: Results of a Phase IV open-label randomized controlled study (the SMART study). *Diabetes Obes Metab* 2017; **19**(11): 1513-20.

6. Pratley RE, Eldor R, Raji A, et al. Ertugliflozin plus sitagliptin versus either individual agent over 52 weeks in patients with type 2 diabetes mellitus inadequately controlled with metformin: The VERTIS FACTORIAL randomized trial. *Diabetes Obes Metab* 2018; **20**(5): 1111-20.

7. Rosenstock J, Perkovic V, Johansen OE, et al. Effect of Linagliptin vs Placebo on Major Cardiovascular Events in Adults With Type 2 Diabetes and High Cardiovascular and Renal Risk: The CARMELINA Randomized Clinical Trial. *JAMA* 2019; **321**(1): 69-79.

8. Shankar RR, Inzucchi SE, Scarabello V, et al. A randomized clinical trial evaluating the efficacy and safety of the once-weekly dipeptidyl peptidase-4 inhibitor omarigliptin in patients with type 2 diabetes inadequately controlled on metformin monotherapy. *Curr Med Res Opin* 2017; **33**(10): 1853-60.

9. Lee SH, Gantz I, Round E, et al. A randomized, placebo-controlled clinical trial evaluating the safety and efficacy of the once-weekly DPP-4 inhibitor omarigliptin in patients with type 2 diabetes mellitus inadequately controlled by glimepiride and metformin. *BMC Endocr Disord* 2017; **17**(1): 70.

10. Gantz I, Okamoto T, Ito Y, et al. A randomized, placebo- and sitagliptin-controlled trial of the safety and efficacy of omarigliptin, a once-weekly dipeptidyl peptidase-4 inhibitor, in Japanese patients with type 2 diabetes. *Diabetes Obes Metab* 2017; **19**(11): 1602-9.

11. Gantz I, Chen M, Suryawanshi S, et al. A randomized, placebo-controlled study of the cardiovascular safety of the once-weekly DPP-4 inhibitor omarigliptin in patients with type 2 diabetes mellitus. *Cardiovasc Diabetol* 2017; **16**(1): 112.

12. Handelsman Y, Lauring B, Gantz I, et al. A randomized, double-blind, non-inferiority trial evaluating the efficacy and safety of omarigliptin, a once-weekly DPP-4 inhibitor, or glimepiride in patients with type 2 diabetes inadequately controlled on metformin monotherapy. *Curr Med Res Opin* 2017; **33**(10): 1861-8.

13. Mathieu C, Catrinoiu D, Ranetti AE, et al. Characterization of the Open-Label Lead-In Period of Two Randomized Controlled Phase 3 Trials Evaluating Dapagliflozin, Saxagliptin, and Metformin in Type 2 Diabetes. *Diabetes Ther* 2018; **9**(4): 1703-11.

14. Ji L, Zinman B, Patel S, et al. Efficacy and safety of linagliptin co-administered with low-dose metformin once daily versus high-dose metformin twice daily in treatment-naive patients with type 2 diabetes: a double-blind randomized trial. *Adv Ther* 2015; **32**(3): 201-15.

15. DeFronzo RA, Lewin A, Patel S, et al. Combination of empagliflozin and linagliptin as second-line therapy in subjects with type 2 diabetes inadequately controlled on metformin. *Diabetes Care* 2015; **38**(3): 384-93.

16. Roden M, Merker L, Christiansen AV, et al. Safety, tolerability and effects on cardiometabolic risk factors of empagliflozin monotherapy in drug-naive patients with type 2 diabetes: a double-blind extension of a Phase III randomized controlled trial. *Cardiovasc Diabetol* 2015; **14**: 154.

17. Pan C, Han P, Ji Q, et al. Efficacy and safety of alogliptin in patients with type 2 diabetes mellitus: A multicentre randomized double-blind placebo-controlled Phase 3 study in mainland China, Taiwan, and Hong Kong. *J Diabetes* 2017; **9**(4): 386-95.

18. Rosenstock J, Kahn SE, Johansen OE, et al.CAROLINA Investigators . Effect of Linagliptin vs Glimepiride on Major Adverse Cardiovascular Outcomes in Patients With Type 2 Diabetes: The CAROLINA Randomized Clinical Trial. JAMA 2019;322:1155-66. 10.1001/jama.2019.13772

19. Sheu WH, Gantz I, Chen M, et al. Safety and Efficacy of Omarigliptin (MK-3102), a Novel Once-Weekly DPP-4 Inhibitor for the Treatment of Patients With Type 2 Diabetes. *Diabetes Care* 2015; **38**(11): 2106-14.

20. Wang W, Yang J, Yang G, et al. Efficacy and safety of linagliptin in Asian patients with type 2 diabetes mellitus inadequately controlled by metformin: A multinational 24-week, randomized clinical trial. *J Diabetes* 2016; **8**(2): 229-37.

21. Inagaki N, Watada H, Murai M, et al. Linagliptin provides effective, well-tolerated add-on therapy to pre-existing oral antidiabetic thera py over 1 year in Japanese patients with type 2 diabetes. *Diabetes Obes Metab* 2013; **15**(9): 833-43.

22. Nauck MA, di Domenico M, Patel S, Kobe M, Toorawa R, Woerle HJ. Linagliptin and pioglitazone combination therapy versus monotherapy with linagliptin or pioglitazone: A randomised, double-blind, parallel-group, multinational clinical trial. *Diab Vasc Dis Res* 2016; **13**(4): 286-98.

23. Wang W, Ning G, Ma J, et al. A randomized clinical trial of the safety and efficacy of sitagliptin in patients with type 2 diabetes mellitus inadequately controlled by acarbose alone. *Curr Med Res Opin* 2017; **33**(4): 693-9.

24. Schernthaner G, Gross JL, Rosenstock J, et al. Canagliflozin compared with sitagliptin for patients with type 2 diabetes who do not have adequate glycemic control with metformin plus sulfonylurea: a 52-week randomized trial. *Diabetes Care* 2013; **36**(9): 2508-15.

25. Mosenzon O, Leibowitz G, Bhatt DL, et al. Effect of Saxagliptin on Renal Outcomes in the SAVOR-TIMI 53 Trial. *Diabetes Care* 2017; **40**(1): 69-76.

26. Lavalle-Gonzalez FJ, Januszewicz A, Davidson J, et al. Efficacy and safety of canagliflozin compared with placebo and sitagliptin in patients with type 2 diabetes on background metformin monotherapy: a randomised trial. *Diabetologia* 2013; **56**(12): 2582-92.

27. Moses RG, Round E, Shentu Y, et al. A randomized clinical trial evaluating the safety and efficacy of sitagliptin added to the combination of sulfonylurea and metformin in patients with type 2 diabetes mellitus and inadequate glycemic control. *J Diabetes* 2016; **8**(5): 701-11.

28. Philis-Tsimikas A, Del Prato S, Satman I, et al. Effect of insulin degludec versus sitagliptin in patients with type 2 diabetes uncontrolled on oral antidiabetic agents. *Diabetes Obes Metab* 2013; **15**(8): 760-6.

29. White WB, Cannon CP, Heller SR, et al. Alogliptin after acute coronary syndrome in patients with type 2 diabetes. *N Engl J Med* 2013; **369**(14): 1327-35.

30. Yki-Jarvinen H, Rosenstock J, Dur¨¢n-Garcia S, et al. Effects of adding linagliptin to basal insulin regimen for inadequately controlled type 2 diabetes: a ¡Ý52-week randomized, double-blind study. *Diabetes Care* 2013; **36**(12): 3875-81.

31. Gordon J, McEwan P, Hurst M, Puelles J. The Cost-Effectiveness of Alogliptin Versus Sulfonylurea as Add-on Therapy to Metformin in Patients with Uncontrolled Type 2 Diabetes Mellitus. *Diabetes Ther* 2016; **7**(4): 825-45.

32. Ahr¨¦n B, Johnson SL, Stewart M, et al. HARMONY 3: 104-week randomized, double-blind, placebo- and active-controlled trial assessing the effi cacy and safety of albiglutide compared with placebo, sitagliptin, and glimepiride in patients with type 2 diabetes taking metformin. *Diabetes Care* 2014; **37**(8): 2141-8.

33. McGuire DK, Van de Werf F, Armstrong PW, et al. Association Between Sitagliptin Use and Heart Failure Hospitalization and Related Outcomes in Type 2 Diabetes Mellitus: Secondary Analysis of a Randomized Clinical Trial. *JAMA Cardiol* 2016; **1**(2): 126-35.

34. Barnett AH, Charbonnel B, Donovan M, Fleming D, Chen R. Effect of saxagliptin as add-on therapy in patients with poorly controlled type 2 diabetes on insulin alone or insulin combined with metformin. *Curr Med Res Opin* 2012; **28**(4): 513-23.

35. Henry RR, Staels B, Fonseca VA, et al. Efficacy and safety of initial combination treatment with sitagliptin and pioglitazone--a factorial s tudy. *Diabetes Obes Metab* 2014; **16**(3): 223-30.

36. Horie Y, Hayashi N, Dugi K, Takeuchi M. Design, statistical analysis and sample size calculation of a phase IIb/III study of linagliptin versus voglibose and placebo. *Trials* 2009; **10**: 82.

37. Goke B, Gallwitz B, Eriksson JG, Hellqvist A, Gause-Nilsson I. Saxagliptin vs. glipizide as add-on therapy in patients with type 2 diabetes mellitus inadequately controlled on metformin alone: long-term (52-week) extension of a 52-week randomised controlled trial. *Int J Clin Pract* 2013; **67**(4): 307-16.

38. Vilsboll T, Rosenstock J, Yki-J?rvinen H, et al. Efficacy and safety of sitagliptin when added to insulin therapy in patients with type 2 diabetes. *Diabetes Obes Metab* 2010; **12**(2): 167-77.

39. Jadzinsky M, Pfutzner A, Paz-Pacheco E, et al. Saxagliptin given in combination with metformin as initial therapy improves glycaemic control in patients with type 2 diabetes compared with either monotherapy: a randomized controlled trial. *Diabetes Obes Metab* 2009; **11**(6): 611-22.

40. Mohan V, Yang W, Son HY, et al. Efficacy and safety of sitagliptin in the treatment of patients with type 2 diabetes in China, India, and Korea. *Diabetes Res Clin Pract* 2009; **83**(1): 106-16.

41. Pratley RE, Kipnes MS, Fleck PR, Wilson C, Mekki Q, Alogliptin Study G. Efficacy and safety of the dipeptidyl peptidase-4 inhibitor alogliptin in patients with type 2 diabetes inadequately controlled by glyburide monotherapy. *Diabetes Obes Metab* 2009; **11**(2): 167-76.

42. Nauck MA, Ellis GC, Fleck PR, Wilson CA, Mekki Q, Alogliptin Study G. Efficacy and safety of adding the dipeptidyl peptidase-4 inhibitor alogliptin to metformin therapy in patients with type 2 diabetes inadequately controlled with metformin monotherapy: a multicentre, randomised, double-blind, placebo-controlled study. *Int J Clin Pract* 2009; **63**(1): 46-55.

43. Rosenstock J, Rendell MS, Gross JL, Fleck PR, Wilson CA, Mekki Q. Alogliptin added to insulin therapy in patients with type 2 diabetes reduces HbA(1C) without causing weight gain or increased hypoglycaemia. *Diabetes Obes Metab* 2009; **11**(12): 1145-52.

44. DeFronzo RA, Hissa MN, Garber AJ, et al. The efficacy and safety of saxagliptin when added to metformin therapy in patients with inadequately controlled type 2 diabetes with metformin alone. *Diabetes Care* 2009; **32**(9): 1649-55.

45. Rosenstock J, Aguilar-Salinas C, Klein E, et al. Effect of saxagliptin monotherapy in treatment-naive patients with type 2 diabetes. *Curr Med Res Opin* 2009; **25**(10): 2401-11.

46. Goldstein BJ, Feinglos MN, Lunceford JK, Johnson J, Williams-Herman DE, Sitagliptin 036 Study G. Effect of initial combination therapy with sitagliptin, a dipeptidyl peptidase-4 inhibitor, and metformin on glycemic control in patients with type 2 diabetes. *Diabetes Care* 2007; **30**(8): 1979-87.

47. Rosenstock J, Brazg R, Andryuk PJ, Lu K, Stein P, Sitagliptin Study G. Efficacy and safety of the dipeptidyl peptidase-4 inhibitor sitagliptin added to ongoing pioglitazone therapy in patients with type 2 diabetes: a 24-week, multicenter, randomized, double-blind, placebo -controlled, parallel-group study. *Clin Ther* 2006; **28**(10): 1556-68.

1. Rosenstock J, Inzucchi SE, Seufert J, Fleck PR, Wilson CA, Mekki Q. Initial combination therapy with alogliptin and pioglitazone in drug-naïve patients with type 2 diabetes. Diabetes Care 2010;33:2406-8. 10.
2. DeFronzo RA, Burant CF, Fleck P, Wilson C, Mekki Q, Pratley RE. Efficacy and tolerability of the DPP-4 inhibitor alogliptin combined with pioglitazone, in metformin-treated patients with type 2 diabetes. J Clin Endocrinol Metab 2012;97:1615-22.
3. Rosenstock J, Wilson C, Fleck P. Alogliptin versus glipizide monotherapy in elderly type 2 diabetes mellitus patients with mild hyperglycaemia: a prospective, double-blind, randomized, 1-year study. Diabetes Obes Metab 2013;15:906-14.
4. Pratley RE, Fleck P, Wilson C. Efficacy and safety of initial combination therapy with alogliptin plus metformin versus either as monotherapy in drug-naïve patients with type 2 diabetes: a randomized, double-blind, 6-month study. Diabetes Obes Metab 2014;16:613-21.
5. Gomis R, Espadero RM, Jones R, Woerle HJ, Dugi KA. Efficacy and safety of initial combination therapy with linagliptin and pioglitazone in patients with inadequately controlled type 2 diabetes: a randomized, double-blind, placebo-controlled study. Diabetes Obes Metab 2011;13:653-61.
6. Owens DR, Swallow R, Dugi KA, Woerle HJ. Efficacy and safety of linagliptin in persons with type 2 diabetes inadequately controlled by a combination of metformin and sulphonylurea: a 24-week randomized study. Diabet Med 2011;28:1352-61.
7. Gallwitz B, Rosenstock J, Rauch T, et al.. 2-year efficacy and safety of linagliptin compared with glimepiride in patients with type 2 diabetes inadequately controlled on metformin: a randomised, double-blind, non-inferiority trial. Lancet 2012;380:475-83.
8. Lewin AJ, Arvay L, Liu D, Patel S, von Eynatten M, Woerle HJ. Efficacy and tolerability of linagliptin added to a sulfonylurea regimen in patients with inadequately controlled type 2 diabetes mellitus: an 18-week, multicenter, randomized, double-blind, placebo-controlled trial. Clin Ther 2012;34:1909-19.e15.
9. Haak T, Meinicke T, Jones R, Weber S, von Eynatten M, Woerle HJ. Initial combination of linagliptin and metformin in patients with type 2 diabetes: efficacy and safety in a randomised, double-blind 1-year extension study. Int J Clin Pract 2013;67:1283-93.
10. Laakso M, Rosenstock J, Groop PH, et al.. Treatment with the dipeptidyl peptidase-4 inhibitor linagliptin or placebo followed by glimepiride in patients with type 2 diabetes with moderate to severe renal impairment: a 52-week, randomized, double-blind clinical trial. Diabetes Care 2015;38:e15-7.
11. Yang W, Xu X, Lei T, et al.. Efficacy and safety of linagliptin as add-on therapy to insulin in Chinese patients with type 2 diabetes mellitus: A randomized, double-blind, placebo-controlled trial. Diabetes Obes Metab 2021;23:642-7.
12. Chacra AR, Tan GH, Apanovitch A, Ravichandran S, List J, Chen R, CV181-040 Investigators. Saxagliptin added to a submaximal dose of sulphonylurea improves glycaemic control compared with uptitration of sulphonylurea in patients with type 2 diabetes: a randomised controlled trial. Int J Clin Pract 2009;63:1395-406.
13. Yang W, Pan CY, Tou C, Zhao J, Gause-Nilsson I. Efficacy and safety of saxagliptin added to metformin in Asian people with type 2 diabetes mellitus: a randomized controlled trial. Diabetes Res Clin Pract 2011;94:217-24.
14. Frederich R, McNeill R, Berglind N, Fleming D, Chen R. The efficacy and safety of the dipeptidyl peptidase-4 inhibitor saxagliptin in treatment-naïve patients with type 2 diabetes mellitus: a randomized controlled trial. Diabetol Metab Syndr 2012;4:36.
15. Hermans MP, Delibasi T, Farmer I, et al. Effects of saxagliptin added to sub-maximal doses of metformin compared with uptitration of metformin in type 2 diabetes: the PROMPT study. Curr Med Res Opin 2012;28:1635-45.
16. Schernthaner G, Durán-Garcia S, Hanefeld M, et al. Efficacy and tolerability of saxagliptin compared with glimepiride in elderly patients with type 2 diabetes: a randomized, controlled study (GENERATION). Diabetes Obes Metab 2015;17:630-8.
17. Chen Y, Liu X, Li Q, et al. Saxagliptin add-on therapy in Chinese patients with type 2 diabetes inadequately controlled by insulin with or without metformin: Results from the SUPER study, a randomized, double-blind, placebo-controlled trial. Diabetes Obes Metab 2018;20:1044-9.
18. Pollock C, Stefánsson B, Reyner D, et al. Albuminuria-lowering effect of dapagliflozin alone and in combination with saxagliptin and effect of dapagliflozin and saxagliptin on glycaemic control in patients with type 2 diabetes and chronic kidney disease (DELIGHT): a randomised, double-blind, placebo-controlled trial. Lancet Diabetes Endocrinol 2019;7:429-41.
19. Nauck MA, Meininger G, Sheng D, Terranella L, Stein PP, Sitagliptin Study 024 Group. Efficacy and safety of the dipeptidyl peptidase-4 inhibitor, sitagliptin, compared with the sulfonylurea, glipizide, in patients with type 2 diabetes inadequately controlled on metformin alone: a randomized, double-blind, non-inferiority trial. Diabetes Obes Metab 2007;9:194-205.
20. Barzilai N, Guo H, Mahoney EM, et al. Efficacy and tolerability of sitagliptin monotherapy in elderly patients with type 2 diabetes: a randomized, double-blind, placebo-controlled trial. Curr Med Res Opin 2011;27:1049-58.
21. Arjona Ferreira JC, Marre M, Barzilai N, et al. Efficacy and safety of sitagliptin versus glipizide in patients with type 2 diabetes and moderate-to-severe chronic renal insufficiency. Diabetes Care 2013;36:1067-73.
22. Dobs AS, Goldstein BJ, Aschner P, et al. Efficacy and safety of sitagliptin added to ongoing metformin and rosiglitazone combination therapy in a randomized placebo-controlled 54-week trial in patients with type 2 diabetes. J Diabetes 2013;5:68-79.
23. Roden M, Weng J, Eilbracht J, et al. EMPA-REG MONO trial investigators . Empagliflozin monotherapy with sitagliptin as an active comparator in patients with type 2 diabetes: a randomised, double-blind, placebo-controlled, phase 3 trial. Lancet Diabetes Endocrinol 2013;1:208-19.
24. Ferrannini E, Berk A, Hantel S, et al. Long-term safety and efficacy of empagliflozin, sitagliptin, and metformin: an active-controlled, parallel-group, randomized, 78-week open-label extension study in patients with type 2 diabetes. Diabetes Care 2013;36:4015-21.
25. Terauchi Y, Yamada Y, Ishida H, et al.. Efficacy and safety of sitagliptin as compared with glimepiride in Japanese patients with type 2 diabetes mellitus aged ≥ 60 years (START-J trial). Diabetes Obes Metab 2017;19:1188-92.
26. Matthews DR, Paldánius PM, Proot P, Chiang Y, Stumvoll M, Del Prato S, VERIFY study group. Glycaemic durability of an early combination therapy with vildagliptin and metformin versus sequential metformin monotherapy in newly diagnosed type 2 diabetes (VERIFY): a 5-year, multicentre, randomised, double-blind trial. Lancet 2019;394:1519-29.
27. Rosenstock J, Perl S, Johnsson E, García-Sánchez R, Jacob S. Triple therapy with low-dose dapagliflozin plus saxagliptin versus dual therapy with each monocomponent, all added to metformin, in uncontrolled type 2 diabetes. Diabetes Obes Metab 2019;21:2152-62.
28. Ledesma G, Umpierrez GE, Morley JE, et al.. Efficacy and safety of linagliptin to improve glucose control in older people with type 2 diabetes on stable insulin therapy: A randomized trial. Diabetes Obes Metab 2019;21:2465-73.
