# Supplementary material for: Association of the gallbladder or biliary diseases with dipeptidyl peptidase 4 inhibitors in patients with type 2 diabetes: a meta-analysis of randomized controlled trials
Source: Diabetol Metab Syndr. 2022 Oct 21;14:153. doi: 10.1186/s13098-022-00924-8 (PMC9585736; doi:10.1186/s13098-022-00924-8)
Supplement: Supplementary file 4 — Additional file 4. Overall heterogeneity levels for each outcomes. [file 13098_2022_924_MOESM4_ESM.doc]

**Supplement Appendix 4. Overall heterogeneity levels for each outcomes.**

| Comparators: placebo or active drug | | |
| --- | --- | --- |
|  | I^2^ | P value |
| cholecystitis | 0.0% | 0.992 |
| cholangitis | 0.0% | 0.954 |
| cholelithiasis | 0.0% | 0.997 |
| bile duct stone | 0.0% | 0.912 |
| biliary colic | 0.0% | 0.680 |
| overall gallbladder or biliary disease | 0.0% | 0.937 |
| Comparators: placebo | | |
|  | I^2^ | P value |
| cholecystitis | 0.0% | 0.967 |
| cholangitis | 0.0% | 0.827 |
| cholelithiasis | 0.0% | 0.992 |
| bile duct stone | 0.0% | 0.902 |
| biliary colic | 0.0% | 0.442 |
| overall gallbladder or biliary disease | 0.0% | 0.213 |
