# Supplementary material for: Association of the gallbladder or biliary diseases with dipeptidyl peptidase 4 inhibitors in patients with type 2 diabetes: a meta-analysis of randomized controlled trials
Source: Diabetol Metab Syndr. 2022 Oct 21;14:153. doi: 10.1186/s13098-022-00924-8 (PMC9585736; doi:10.1186/s13098-022-00924-8)
Supplement: Supplementary file 5 — Additional file 5. Funnel plot for the meta-analysis of the association between DPP4i and risk of gallbladder or biliary diseases. [file 13098_2022_924_MOESM5_ESM.doc]

Supplement Appendix 5. Funnel plot for the meta-analysis of the association between DPP4i and risk of gallbladder or biliary diseases
